# Dysregulated cross-talk between alveolar epithelial cells and stromal cells in idiopathic pulmonary fibrosis reduces epithelial regenerative capacity

**DOI:** 10.3389/fmed.2023.1182368

**Published:** 2023-08-09

**Authors:** Marissa Wisman, Mehmet Nizamoglu, Jacobien A. Noordhoek, Wim Timens, Janette K. Burgess, Irene H. Heijink

**Affiliations:** ^1^University of Groningen, University Medical Center Groningen, Department of Pathology and Medical Biology, Groningen, Netherlands; ^2^University of Groningen, University Medical Center Groningen, Groningen Research Institute for Asthma and COPD (GRIAC), Groningen, Netherlands; ^3^University of Groningen, University Medical Center Groningen, Department of Pulmonology, Groningen, Netherlands; ^4^University of Groningen, University Medical Center Groningen, W.J. Kolff Institute for Biomedical Engineering and Materials Science-FB41, Groningen, Netherlands

**Keywords:** idiopathic pulmonary fibrosis, alveolar epithelial repair, epithelial mesenchymal cross-talk, epithelial regeneration, lung organoids

## Abstract

In idiopathic pulmonary fibrosis (IPF) constant epithelial micro-injury and aberrant interactions within the stromal micro-environment lead to abnormal alveolar repair and fibrosis. We hypothesized that alveolar epithelial regenerative responses in IPF are impaired due to disturbed crosstalk between epithelial cells and their stromal niche. We established organoid cultures from unfractionated suspensions and isolated EpCAM^+^ cells from distal lung tissue of patients with and without IPF. We observed significantly more organoids being formed from unfractionated suspensions compared to isolated EpCAM^+^ cell cultures, indicating the presence of supportive cells in the unfractionated suspensions. Importantly, lower organoid numbers were observed in unfractionated cultures from IPF lungs compared to non-IPF lungs. This difference was not found when comparing organoid formation from isolated EpCAM^+^ cells alone between IPF and non-IPF groups, suggesting that crosstalk between the supportive population and epithelial cells is impaired in lungs from IPF patients. Additionally, organoids grown from IPF lung-derived cells were larger in size compared to those from non-IPF lungs in both unfractionated and EpCAM^+^ cultures, indicating an intrinsic abnormality in epithelial progenitors from IPF lungs. Together, our observations suggest that dysregulated crosstalk between alveolar progenitor cells and the stromal niche affects the regenerative capacity, potentially contributing to alveolar impairment in IPF.

## Introduction

Idiopathic Pulmonary Fibrosis (IPF) is a progressive lung disease characterized by aberrant repair responses in the alveoli, leading to fibrosis and rapid lung function decline. A high mortality rate, while having no cure available, illustrates the urgent need to understand IPF pathogenesis to identify new therapeutic strategies (1). The origin of the disease is still unknown, but ongoing alveolar epithelial micro-injury and aberrant interactions within the stromal micro-environment are thought to induce the abnormal alveolar regeneration and tissue repair (2). The crosstalk between epithelial cells and their stromal niche composed of supportive cells and extracellular matrix (ECM) is critical for alveolar repair (3). The stromal compartment includes fibroblasts, mesenchymal stromal cells, macrophages and endothelial cells, as well as ECM (4). Emerging data suggests that stromal alterations in IPF lead to inadequate alveolar epithelial regeneration (5).

In this study we hypothesized that the crosstalk between alveolar epithelial cells and other cell types present in the fibrotic micro-environment of the lung is disrupted, resulting in reduced regenerative capacity of alveolar epithelial progenitors derived from IPF patients. We studied epithelial regenerative potential using an organoid model where alveolar epithelial progenitors were seeded into a 3-dimensional (3D) hydrogel (Matrigel) with stromal cells to recapitulate critical aspects of alveolar regeneration (6), including self-organization into 3D structures, proliferation and differentiation.

## Materials and methods

### Subjects

Parenchymal lung tissue was derived from 4 non-IPF donors undergoing tumor resection surgery and 4 IPF donors undergoing lung transplantation surgery (characteristics are shown in Table 1). Tissue derived from the non-IPF donors was taken from anatomically normal tissue as assessed by experienced pathologists, as far away from the tumor region as possible. This protocol was consistent with the Research Code of the University Medical Center Groningen^1^ and national ethical and professional guidelines (Code of conduct—in Dutch)^2^.

**Table 1:** Characteristics of donors included in the study.

	Non-IPF (*n* = 4)	IPF (*n* = 4)	*p* Value
Sex	3 M/1F	3 M/1F	>0.999^#^
Smoking history			0.0285^#^
Former	3	0	
Never	1	4	
Age [median (min–max)]	55 (36–58)	61 (27–68)	0.3143^†^
FEV1% (Pred.) [median (min–max)]	96.5 (70–111)	42.0 (17–64)	0.0286^†^

# Indicates *p* value as assessed by the Chi-square test.

† Indicates *p* value as assessed by the Mann–Whitney test.

IPF: Idiopathic pulmonary fibrosis, FEV1 (pred.): Forced expiratory volume in 1 s (predicted).

### Tissue dissociation

Unfractionated cell suspensions were obtained from parenchymal lung tissue, from which larger airways (> 2 mm) were removed if found during visual inspection, as previously described by Kruk et al. (7). Briefly, lung tissue was cut into small sections (1 cm^3^) and treated overnight at 4°C with Trypsin/EDTA (0.25%; Gibco, Waltham, MA, United States), supplemented with 1% penicillin (100 U/mL)/streptomycin (100 μg/mL; P/S; Gibco), Collagenase A (2 mg/mL; Roche, Basel, Switzerland) and DNase (0.04 mg/mL, Sigma-Aldrich, Burlington, VT, United States). Subsequently, EpCAM (CD326)^+^ epithelial progenitors were isolated from this unfractionated cell suspensions from 3 non-IPF and 4 IPF donor (tissue from 1 non-IPF donor did not yield sufficient cell numbers) by negative selection for CD31 and CD45 to deplete endothelial cells and hematopoietic cells (8), followed by a positive selection for CD326 using magnetic beads (human anti-CD31, human anti-CD45, human anti-CD326; Miltenyi Biotec, Bergisch Gladback, Germany) according to manufacturer’s instructions. The cell suspensions were then resuspended in Small Airway Growth Medium (SAGM; PromoCell, Heidelberg, Germany), counted manually and kept on ice until use.

### Organoid culture

MRC-5 human fetal lung fibroblasts (ATCC, Manassas, VA, United States) were cultured in Ham’s F12 medium (Gibco) containing 10% fetal bovine serum (FBS; Sigma-Aldrich) and 1% P/S. When confluent, cells were treated with Mitomycin C (0.01 mg/mL; Sigma-Aldrich) for 2 h to inhibit proliferation. Subsequently, cells were trypsinized and resuspended in SAGM, counted manually and kept on ice until use. To generate organoids, unfractionated lung cell suspensions (10.000 cells) or EpCAM^+^ epithelial cells (5.000 or 10.000 cells depending on the yield from the available tissue) were mixed in a 1:1 ratio with MRC-5 cells. This cell mixture, diluted 2:1 with SAGM, was seeded in 100 μL Matrigel (8.4 mg/mL; Corning, New York, MA, United States) on top of 6.5 mm Transwell inserts (0.4 μm pore size; Corning) and cultured for 7 days in SAGM medium containing 1% FBS and 1% P/S in the basolateral compartment. At day 7, images of the organoid cultures were taken using a Nikon Eclipse Ti-E microscope (Brightfield; Nikon Instruments Europe, Amsterdam, Netherlands) and the organoid numbers and organoid size (diameter) were quantified manually throughout the full z axis of the gel using Nikon Eclipse Ti software (Nikon Instruments) to assess the size of each individual organoid. For the calculations of the diameter, for each organoid the measurement plane was set in the middle of the spheres. To calculate the organoid forming efficiency, for unfractionated cultures organoid numbers were corrected for the number of EpCAM^+^ cells isolated from the tissue of that donor and the efficiency of EpCAM^+^ cultures was corrected for the input of epithelial cells (5.000 or 10.000).

### Immunohistochemistry

Cytospin slides were prepared from the unfractionated cell suspension directly after dissociation, for immunohistochemical analyses. Slides were fixed with acetone (Merck, Darmstadt, Germany) and blocked with 5% bovine serum albumin (BSA; Sigma-Aldrich)/1 × Phosphate Buffered Saline (PBS; Gibco) and 0.25% Hydrogen Peroxide (Merck)/1 × PBS, both for 30 min at room temperature (RT). The slides were stained overnight with primary antibody solutions for EpCAM (1:1000; Invitrogen, Massachusetts, United States), endothelial cell marker CD31 (1:100; Immunotools, Friesoythe, Germany), stromal cell marker CD90 (1:100; Biolegend, San Diego, CA, United States), or macrophage marker CD68 (1:100; Agilent Dako, Santa Clara, CA, United States) in 1% BSA/0.1% Triton-X (Merck)/1 × PBS. The next day, slides were washed with 1 × PBS and incubated for 1 h at RT with a secondary antibody solution (1:50 Rabbit-anti Mouse (Agilent Dako) in 1% BSA/0.1% Triton-X/1 × PBS). After washing the slides with 1 × PBS, they were incubated for1 hour at RT with a tertiary antibody solution (1:50 Goat-anti Rabbit (Agilent Dako) in 1% BSA/0.1% Triton-X/1 × PBS) where after color was developed using a NovaRed Substrate kit (Vector labs, Newark, CA, United States) according to the company’s manual. Hematoxylin solution (Sigma-Aldrich) was used to counterstain the nuclei. From a total cell count of at minimum 150 cells, positive stained cells were counted manually. The presence of bronchiolization in IPF lung samples was identified in hematoxylin and eosin (H&E)—stained sections from paraffin embedded lung tissue taken near the site of tissue used for the organoid cultures.

### Statistics

One-way ANOVA with Šídák’s multiple comparisons test was used to assess for statistical differences after testing the normality of the data with Q–Q plots and Shapiro–Wilk test. In case of non-parametric data, the Mann–Whitney test was used for comparisons of two groups and the Kruskal–Wallis test for comparison of more than two groups. *p* < 0.05 was considered statistically significant.

## Results

### IPF lung-derived cells form a smaller number of organoids independent of the number of epithelial cells

**Figure 1:**
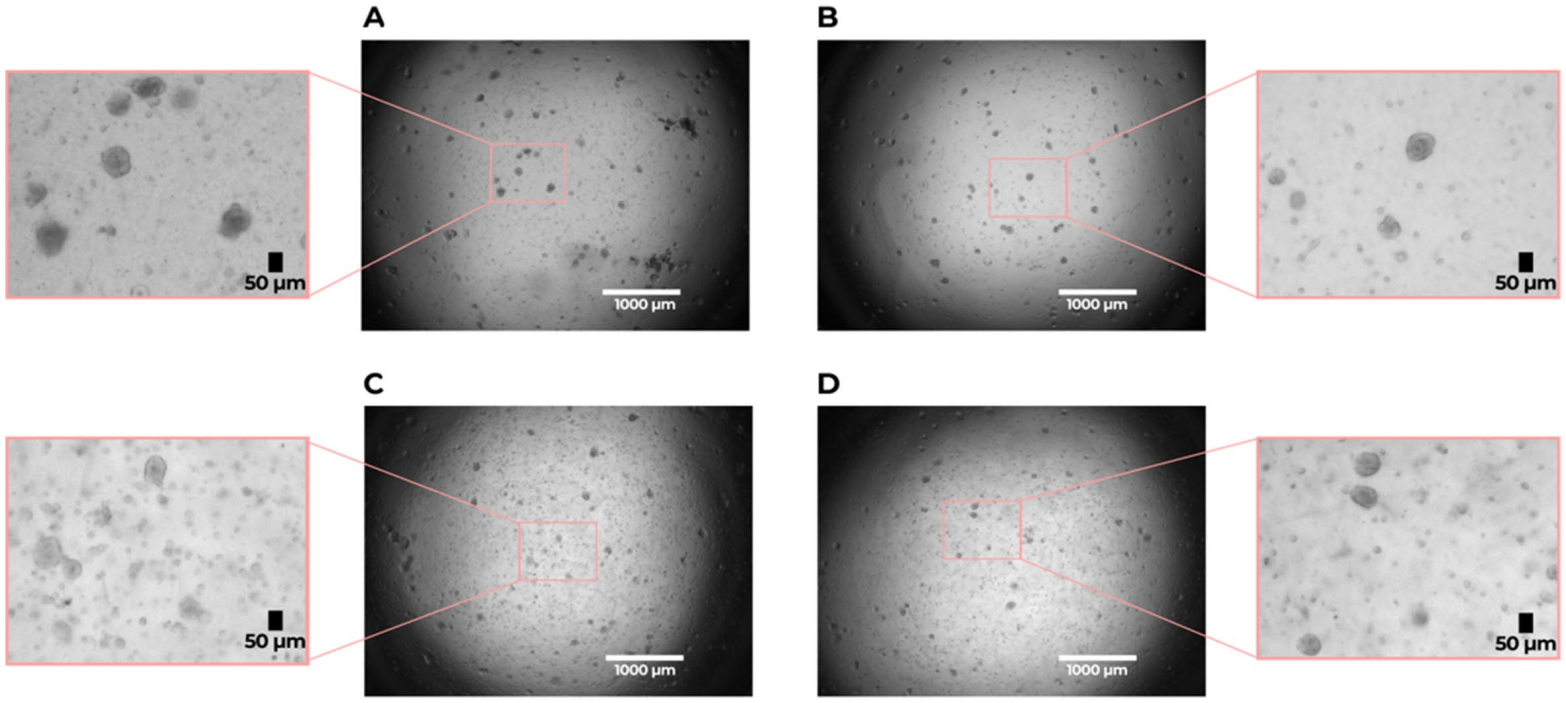
Organoid formation by lung alveolar progenitor cells isolated from idiopathic pulmonary fibrosis (IPF) and non-IPF lungs. **(A)** non-IPF (unfractionated), **(B)** non-IPF EpCAM^+^, **(C)** IPF (unfractionated), **(D)** IPF EpCAM^+^. Brightfield images were taken on day 7 of the organoid cultures with a 2 × magnification. Scale bar: 1,000 μm. The zoomed in sections show representative images of organoids used for analysis with a size of > 50 μm.

**Figure 2:**
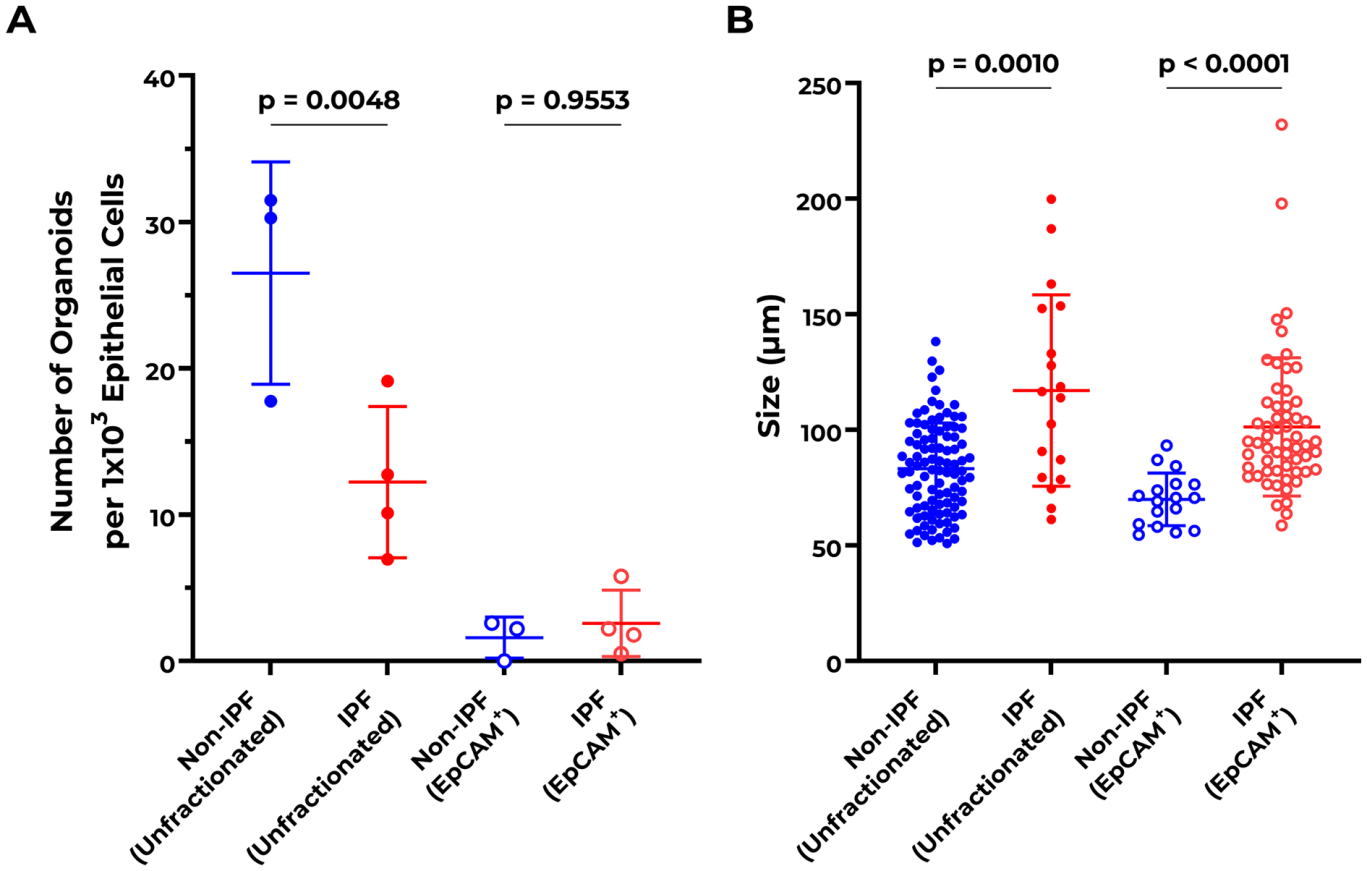
Abnormalities in organoid formation of unfractionated lung cell suspensions from IPF patients. **(A)** Quantification of organoid numbers at day 7 comparing non-IPF (*n* = 3) and IPF (*n* = 4) unfractionated and EpCAM^+^ cultures. Organoid counts of unfractionated suspensions were normalized to the number of isolated EpCAM^+^ cells per donor. EpCAM^+^ cultures were corrected for epithelial cell input during the organoid assay. Means ± SD are indicated. One-way ANOVA with Šídák’s multiple comparisons test was used to assess for statistical differences after testing the data for normality with Q–Q plots and Shapiro–Wilk test. **(B)** Quantification of organoid size distribution at day 7 comparing non-IPF (*n* = 3) and IPF (*n* = 4) unfractionated and EpCAM^+^ cultures. The Kruskal–Wallis test was used to assess for statistical differences.

Both IPF and non-IPF lung-derived cell suspensions were able to form organoids by day 7 (Figure 1). We first compared organoid formation efficiency between the IPF and non-IPF groups in isolated EpCAM^+^ cell populations. We observed that IPF and non-IPF-derived EpCAM^+^ cells were equally well capable of forming organoids, as reflected by both the size of the organoids and the numbers (Figure 2A). Next, we assessed organoid formation efficiency of unfractionated suspensions. The number of EpCAM^+^ cells isolated from IPF lung tissue was significantly lower compared to the non-IPF group (Supplementary Figure 1). Therefore, we normalized the unfractionated cultures for the number of EpCAM^+^ cells isolated from these suspensions, and observed when unfractionated organoid cultures were compared between the groups, that significantly less organoids were formed from IPF lung-derived cells compared to non-IPF lung-derived cells (Figure 2A). We also observed significantly more organoids formed from the unfractionated suspensions compared to isolated EpCAM^+^ cells (Supplementary Figure 2), suggesting the presence of a supportive cell population.

In addition to the number of organoids formed, the size distribution of the organoids was compared between the groups. Notably, IPF-derived cells formed significantly larger organoids compared to the non-IPF group, for both unfractionated (non-IPF organoids mean size 78.45 ± 8.00 μm, IPF organoids mean size 92.70 ± 26.83 μm) and EpCAM^+^ (non-IPF organoids mean size 64.45 ± 1.47 μm, IPF organoids mean size 82.51 ± 14.42 μm) cultures (Figure 2B).

### Cell fractions in unfractionated lung suspensions do not differ significantly between IPF and non-IPF groups

**Figure 3:**
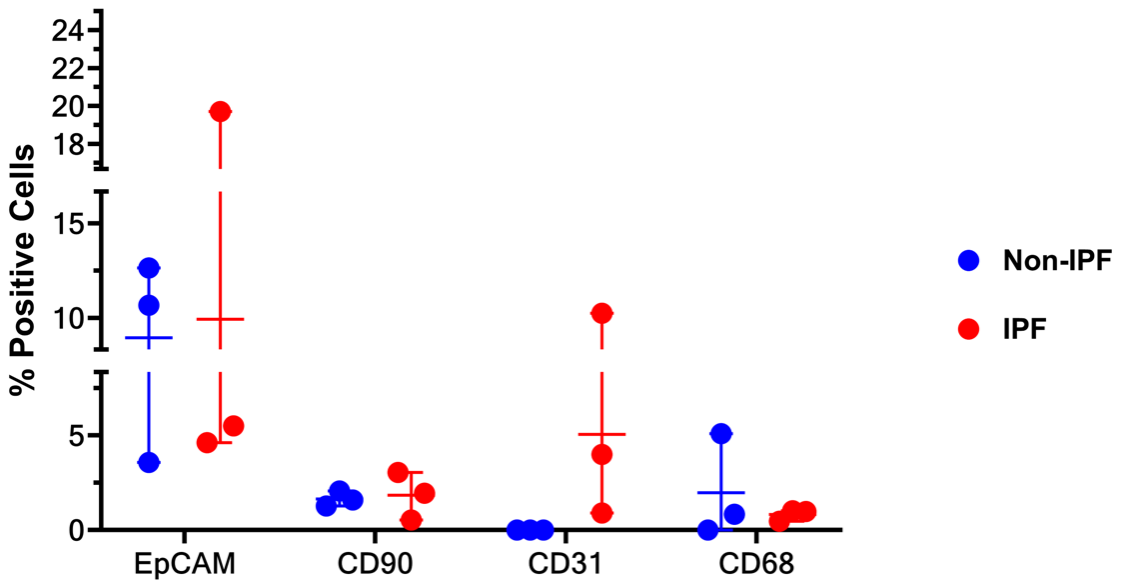
Percentage of cells stained positive for EpCAM (epithelial cells), CD31 (endothelial cells), CD90 (mesenchymal cells), and CD68 (macrophages) in unfractionated cell suspension cytospins from IPF and non-IPF lungs. Means ± SD values are shown. Statistical differences between IPF and non-IPF groups were tested using the unpaired *t*-test after verifying data normality with Q-Q plots and Shapiro–Wilk test.

To investigate whether there were differences in the composition of cell types in the unfractionated suspensions between IPF and non-IPF lungs, cytospins of unfractionated suspensions were stained for several markers. As the stromal compartment of the lungs includes various major cell types, we stained for epithelial cells (EpCAM), stromal cells (CD90), macrophages (CD68) and endothelial cells (CD31), which have all been implicated in IPF pathology (3, 9). We were able to identify CD90^+^, CD68^+^ and CD31^+^ cells in the unfractionated suspensions, notably without positive staining for CD31 in the suspension from non-IPF donors, but we did not observe significant differences in the percentages of each type of cell between the non-IPF and IPF groups (Figure 3).

## Discussion

In this study, we compared the organoid forming efficiency of EpCAM^+^ epithelial cell populations and unfractionated cell suspensions from parenchymal regions of IPF and non-IPF lungs. Our results suggest the presence of a supportive cell population in the unfractionated lung suspensions. Of interest, we observed reduced organoid forming efficiency in the unfractionated suspensions from IPF compared to non-IPF lungs. This difference was not present in organoids formed from isolated EpCAM^+^ cell populations from both IPF and non-IPF lungs, suggesting that dysregulated cross-talk between epithelial cells and other supportive cell populations exists in IPF lungs. In addition to the reduced organoid forming capacity, the organoids derived from IPF lungs were significantly larger compared to non-IPF lung-derived organoids in both unfractionated suspensions and EpCAM^+^ cell populations. To the best of our knowledge, this is the first report of lower organoid forming capacity in cells isolated from parenchymal tissue of lungs of patients with IPF.

Organoid forming efficiency as an indication of regenerative capacity has been demonstrated for several other lung diseases (10). While fewer numbers of epithelial cells were isolated from the lungs from donors with IPF, our results show reduced numbers of organoids in IPF lung-derived cultures in the unfractionated groups independent of the number of epithelial cells. This was not observed in organoid cultures established from isolated EpCAM^+^ epithelial cells. This observation does not align with the current school of thought that suggests that the loss of regenerative capacity (specifically their ability to self-renew) of epithelial cells stems from epithelial–mesenchymal transition or senescence in the epithelial cell population in IPF (11). The comparable organoid forming efficiency seen in isolated EpCAM^+^ cell populations in IPF and non-IPF groups, relative to the starting number of epithelial cells, indicates that the hampered repair capacity does not result directly from defects in the epithelial progenitor cells. Rather, it may be the result of defective interactions between cells within the stromal niche. In line with previous data from our group (7), our results suggest the presence of a supportive cell population in the unfractionated lung suspensions. Although we did not observe significant differences in the proportions of supporting cell types in the isolated cell populations between IPF and non-IPF, stromal cells, endothelial cells and macrophages were found to be present in the unfractionated suspensions. Notably, we did not detect endothelial cells in the non-IPF suspensions. However, because limited tissue was available yielding low cell counts, we could not examine the individual function of the different cell types from the stromal niche. This will be of interest in the future. Furthermore, new multicellular culture models have been used that highlight the importance of a multicellular environment in epithelial regeneration (12–16). It may also be that the fibrotic microenvironment *in vivo* from which these cells were derived has imprinted cells towards different behavior (17); the observed differences in organoid supportive capacity might thus result from dysregulated crosstalk between the epithelial cells and stromal cells dictated by the imprinting from the IPF microenvironment. Nevertheless, we cannot exclude the possibility that an EpCAM^+^ progenitor population, that may be selectively lost upon isolation of EpCAM^+^ cells, contributes to the differences in organoid forming ability of unfractionated cell suspensions and EpCAM^+^ cell populations.

The IPF organoid cultures, both unfractionated and EpCAM^+^ populations, generated larger organoids compared to non-IPF cultures. This could indicate that epithelial progenitors from IPF lungs display intrinsic differences with respect to proliferation or other characteristics that determine organoid size. The morphology of the epithelial cells in tissue sections adjacent to the regions from where we isolated cells from in the lungs of patients with IPF was checked. Previously published reports indicate bronchiolization of the epithelium in the alveolar region in patients with IPF (18), which may be related to the intrinsic differences observed in IPF-derived epithelial cells. When the H&E-stained sections of IPF lung tissue were examined, presence of bronchiolization of the alveolar epithelium was observed in all IPF patients from whom cells were obtained (Supplementary Figure 3). This is relevant as this is indicative of an active bronchiolization process occurring in the alveolar region in the IPF tissues, which is a form of metaplasia, and the larger organoid formation, potentially due to aberrant proliferation or cell transitioning of epithelial progenitors in the IPF organoids, may be a reflection of such metaplasia (19, 20). Of note, bronchiolization does not indicate the presence of more proximal progenitors cells in distal tissue, but is a form of epithelial cell metaplasia indicating that epithelial progenitors in (or in this case coming from) an inflammatory/fibrotic environment are more prone to differentiate towards a proximal phenotype (18). On the other hand, micro-CT studies have demonstrated that small airways might be the origin of honeycomb cysts in IPF, and thus defects in small airway epithelial progenitors may contribute to alveolar abnormalities observed in IPF as well (21). Further studies on whether the IPF organoids contain more or larger cells, or more swelling due to reduced barrier function or more mucus production would be required to investigate the influence of bronchiolization in parenchymal lung tissue, its repair and its role in abnormalities in the organoid forming process.

Our study reporting initial observations on alveolar regeneration in IPF has some limitations that should be recognized. Although the EpCAM^+^ cultures from IPF and non-IPF performed equally in their organoid forming capacity, indicating a similar progenitor population, we did not identify specific alveolar progenitor cells. Further, the lack of differences in the numbers of different cell types in the unfractionated cell populations between the groups may be a consequence of the isolation method, which has been optimized for alveolar epithelial cells and not for other cell types. In addition, the generated cell counts may be an underestimation of the total numbers of cells present in the lung tissue as several of the surface markers are known to be sensitive to cleavage by the enzymatic treatment used to isolate cells. Nevertheless, sufficient cell numbers have been isolated in order to support organoid formation effectively, as indicated by our current findings. Further characterization of isolated cell fractions and investigation of the involvement of cellular interactions during the initiation of organoid formation will be of interest in future studies, but was outside of the scope of this discovery study. Moreover, this was not feasible as receiving donor material from IPF and non-IPF patients is very rare, donors need to be matched based on their detailed characterization, and the amount of tissue available is limited.

As mentioned above, the origin of larger organoids from IPF donors requires further investigation. The finding that the organoid forming efficiency differs between IPF and non-IPF lung-derived organoid cultures from unfractionated suspensions, but not isolated epithelial progenitors, suggests that altered interactions between different cell types derived from IPF lungs are responsible for the aberrations in the initiation phase of the organoid formation. Previous research has shown that WNT-signaling influences the ability of epithelial progenitor cells to self-renew and form organoids (22). In mice it has been shown that epithelial progenitors reside in a stromal niche that provides WNT signals to maintain their stemness (23). Thus, we speculate that the WNT signaling pathway might be dysregulated in IPF-derived organoids due to the disturbed crosstalk between epithelial progenitors and stromal cell types. Further studies are needed to investigate whether alterations in the release of WNT ligands from stromal cells, endothelial cells and/or macrophages or in their interaction with epithelial cells occur in IPF.

## Conclusion

Overall, our observations indicate that aberrant crosstalk between (alveolar) epithelial cells and stromal cells changes the regenerative capacity of alveolar progenitors in IPF. This dysregulation may contribute to the abnormal alveolar repair in IPF, partly leading to bronchial and squamous metaplasia. In addition, this disturbance might also contribute to an abnormal alveolar micro-environment and ECM interactions in the lung in IPF. Further investigations regarding the contribution of specific stromal cells in epithelial regeneration will be necessary to understand the influence of aberrant alveolar repair in IPF pathophysiology. It will be of interest to investigate the behavior of metaplastic bronchial epithelial cells in culture systems like organoids to provide further insight into abnormal alveolar repair in IPF. Future research on the interaction of different epithelial cell types along with studying the communication between epithelial and stromal niche cells through excreted mediators/growth factors in multicellular 3D systems will deepen our understanding of IPF pathophysiology.

## Data availability statement

The raw data supporting the conclusions of this article will be made available by the authors, without undue reservation.

## Author contributions

MW, MN, JB, and IH conceived and designed the study. MW, MN, and JN performed experiments. MW and MN analyzed the data and drafted the manuscript. MW, MN, WT, JB, and IH revised and edited the manuscript. All authors contributed to the article and approved the submitted version.

## Conflict of interest

The authors declare that the research was conducted in the absence of any commercial or financial relationships that could be construed as a potential conflict of interest.

## Publisher’s note

All claims expressed in this article are solely those of the authors and do not necessarily represent those of their affiliated organizations, or those of the publisher, the editors and the reviewers. Any product that may be evaluated in this article, or claim that may be made by its manufacturer, is not guaranteed or endorsed by the publisher.

## Footnotes

^1^ https://www.umcg.nl/documents/770534/2183586/umcg-research-code-2018-en.pdf/9680a460-3feb-543d-7d58-bc9d4f7277de?t=1614951313016

^2^ https://www.coreon.org/gedragscode-gezondheidsonderzoek
