# Supplementary material for: Dysregulated cross-talk between alveolar epithelial cells and stromal cells in idiopathic pulmonary fibrosis reduces epithelial regenerative capacity
Source: Front Med (Lausanne). 2023 Aug 9;10:1182368. doi: 10.3389/fmed.2023.1182368 (PMC10446880; doi:10.3389/fmed.2023.1182368)
Supplement: Supplementary file 1 [file Data_Sheet_1.docx]

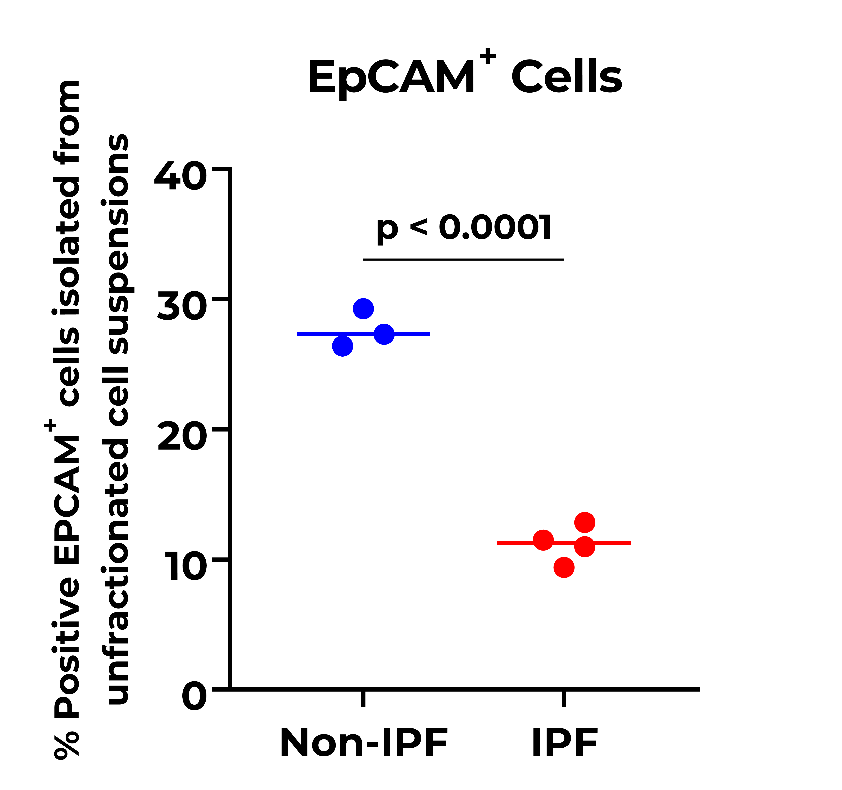


Supplementary Figure 1: Number of EpCAM^+^ positive cells isolated from non-IPF and IPF unfractionated suspensions. N=3 for non-IPF and n=4 for IPF samples. Statistical differences between non-IPF and IPF groups were tested using unpaired t-test after verifying normality with Q-Q plots and Shapiro-Wilk test.


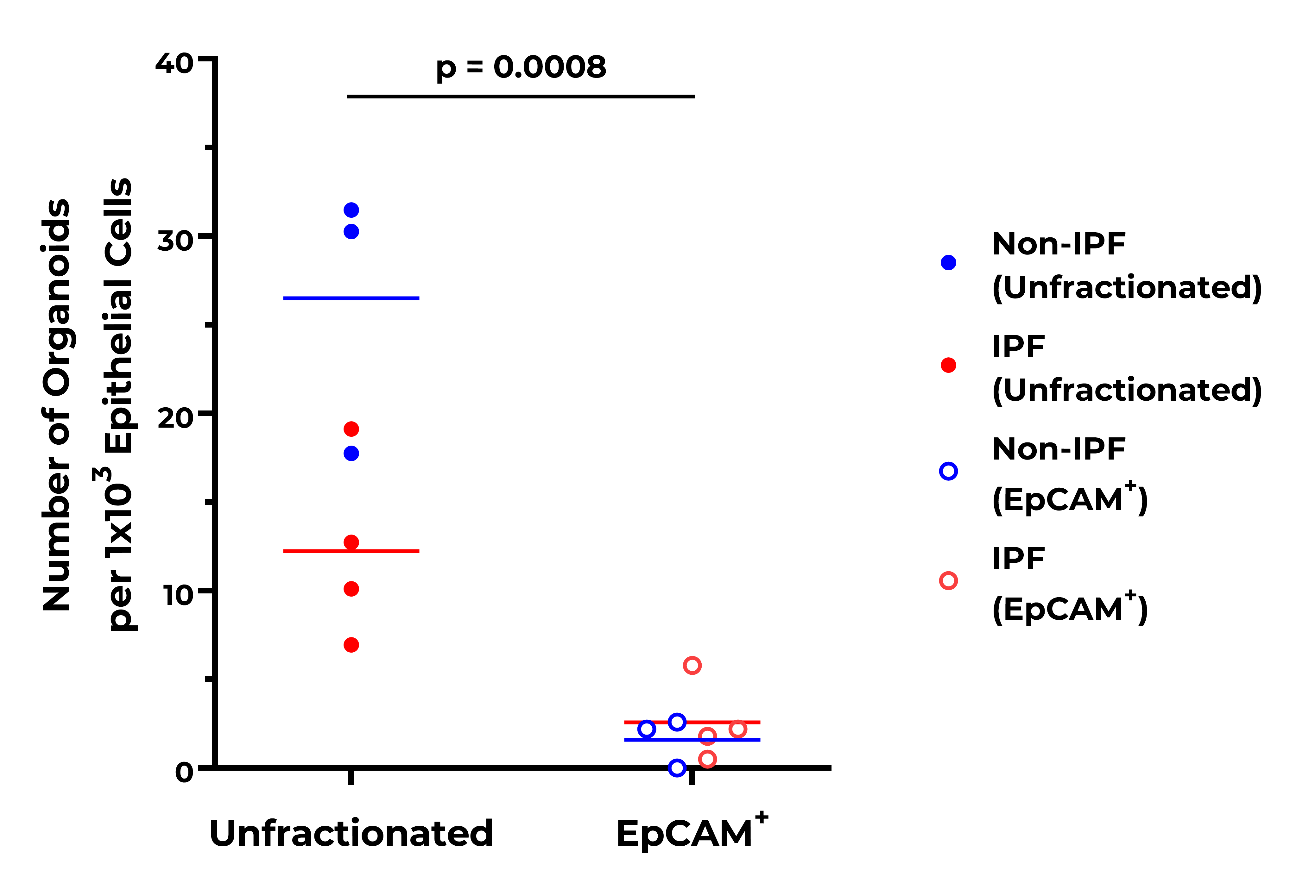


Supplementary Figure 2: Difference in organoid forming efficiency between unfractionated and EpCAM^+^ cell populations of IPF and non-IPF lung-derived epithelial cells. Quantification of organoid numbers at day 7 comparing unfractionated and EpCAM^+^ cultures (n=7 in total). Organoid counts were normalized to the number of EpCAM^+^ cells to correct for differences in epithelial cell input during the organoid assay. Means ±SD are indicated. Statistical differences between groups were tested using the unpaired t-test after verifying normality with Q-Q plots and Shapiro-Wilk test.


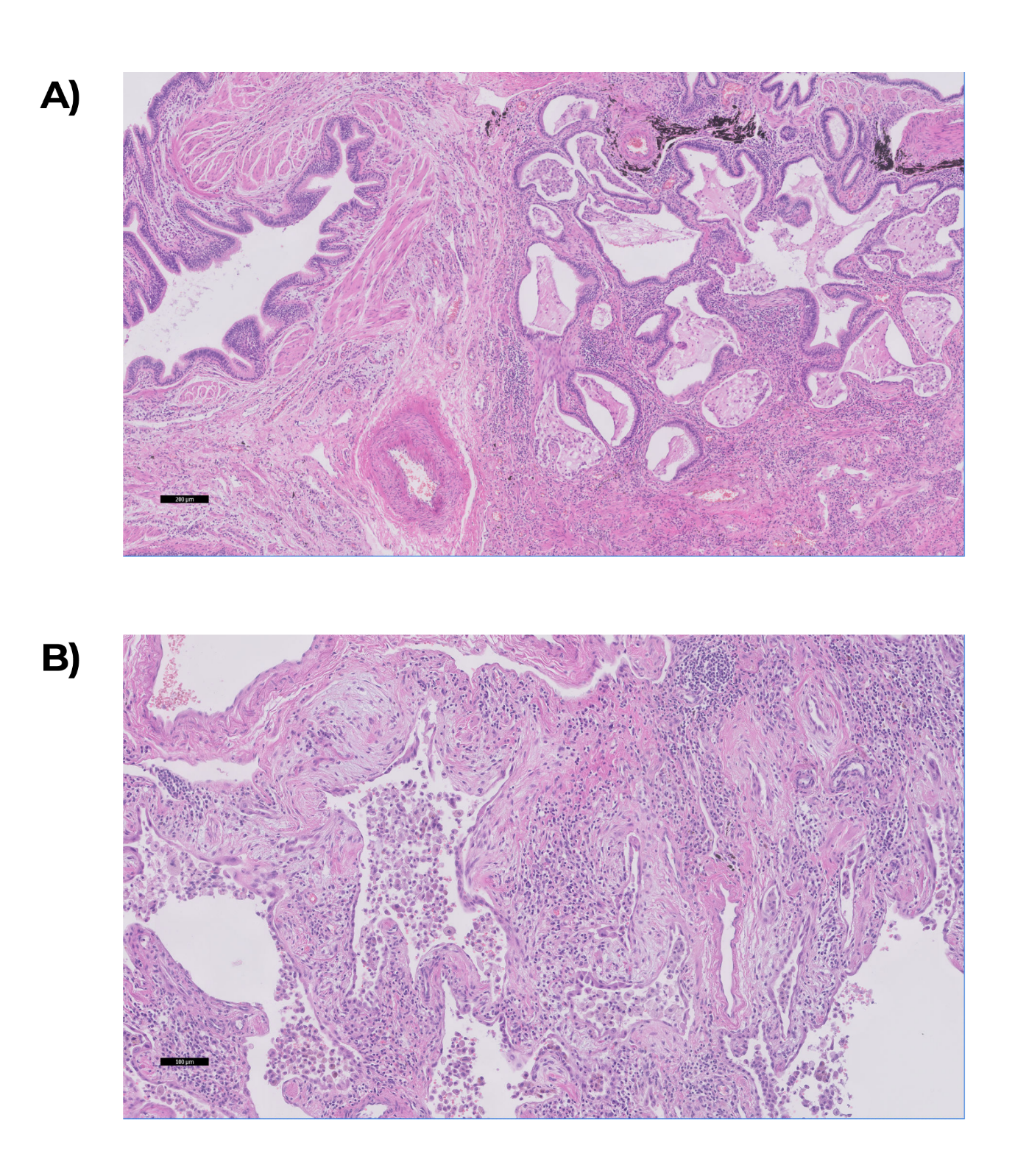


Supplementary Figure 3: Aberrances in alveolar structure in IPF lung tissue. **A)** Representative image showing the presence of bronchiolization (right) adjacent to a pre-existing bronchiole (left) in the lung parenchyma in IPF lung tissue (hematoxylin and eosin, scale bar is 200 μm). **B)** Extensive fibroblastic foci covered by cubic to flat squamous epithelium, bordering a remaining alveoli filled with macrophages (hematoxylin and eosin, scale bar is 100 μm).
